# Estimates of genotypic and phenotypic variance, heritability, and genetic advance of horticultural traits in developed crosses of cowpea (*Vigna unguiculata* [L.] Walp)

**DOI:** 10.3389/fpls.2022.987985

**Published:** 2022-09-27

**Authors:** Haitham E. M. Zaki, Khlode S. A. Radwan

**Affiliations:** ^1^ Horticulture Department, Faculty of Agriculture, Minia University, El-Minia, Egypt; ^2^ Applied Biotechnology Department, University of Technology and Applied Sciences-Sur, Sur, Oman; ^3^ Plant Pathology Department, Faculty of Agriculture, Minia University, El-Minia, Egypt

**Keywords:** cowpea (*Vigna unguiculata* L. Walp.), cowpea crosses, pod traits, yield and yield components, phenotypic variance, genotypic variance, heritability

## Abstract

Cowpea, in addition to being a food and feed crop, plays a key role in sustainable farming. The present study’s goal is to develop new high-yielding cowpea varieties. A Field experiment was carried out across 3 summer seasons and the breeding program included 28 distinct cowpea varieties, out of which five potential parents were selected for this investigation. Local cultivars, i.e., Cream 7 ‘Cr7’, Dokki 331 ‘D331’, Commercial 1 ‘Com1’, and introduced cultivars, i.e., Colossus ‘Col’ and Asian Introduction ‘AI’ were utilized to produce six crosses in two generations apart; F_1_ and F_2_: Col x AI, Col x Com1, Cr7 x AI, Cr7 x Com1, D331 x AI, and D331 x Com1. ‘AI’ and ‘Com1’ were superior in pod length, pod diameter, number of seeds/pod and seeds weight/pod, whereas ‘Col’, ‘Cr7’ and ‘D331’ were superior in seeds yield/plant, number of pods/plant and the least number of aborted ovules/pod. The genotypes/crosses showed greater genotypic variance (GV) than phenotypic variance (PV) for number of pods/plant, pod length, number of seeds/pod, number of aborted ovules/pod, fresh pod weight, seeds weight/pod, and seeds yield/plant. All studied variables showed high heritability (H%) in genotypes/crosses, despite the exception of seeds weight/pod, which ranged from 29.14 in ‘D331’ to 83.7 in F_2_ of Col x Com1. F_2_ plants and their parents’ genotypes showed greater H%. Cr7 x AI developed the most H%, 99.04% for number of pods/plant. D331 x Com1 and Cr7 x AI exhibited moderate H% for fresh pod weight in F_1_, but all other crosses had high H%. F_1_ and F_2_ crosses yielded moderate to high GCV and PCV for number of seeds/pod. Variations in parental genotypes and crossings reflect genetic diversity and the possibility of selection. Crossing with ‘AI,’ and ‘Com1’ genotypes enhanced the performance of the other varieties, ‘Col’, ‘D331’ and ‘Cr7’. Cr7 x Com1 and D331 x AI were selected as the most promising crosses for cowpea breeding programs.

## 1 Introduction

Cowpea (*Vigna unguiculata* L. Walp.) is a diploid species (2n = 22), and it is classified as one of the most pertinent food sources in Africa’s arid and semi-arid regions (Damarany, 1994a; Singh et al., 2002 **;** Singh, 2012 **)**. Cowpea is a leguminous crop with a massive tendency to increase legume production; it is a member of the Phaseoleae (L.) tribe, a family of Leguminosae, and a self-pollinated dicotyledonous crop plant **(** Nameirakpam and Khanna, 2018 **)**. Cowpea cultivars are classified into five species: *unguiculata, sesquipedalis, textles, melanophtalmus, and biflora*. Crop seeds have a high calcium and iron content, as well as carbohydrates and protein, yet they are low in fat (Pavadai et al., 2009; Nwosu and Awa, 2013). Cowpea proteins are high in tryptophan and lysine when compared to other crop plants. As a result, cowpea constitutes an enormous part of the dietary protein, particularly for people living in tropical areas; in Africa, cowpea is alluded to as “poor man’s meat” (Baudoin and Marechal, 2001; Tarawali et al., 2002; Reda et al., 2016). As per FAOSTAT (2019), the world’s cowpea production was approximated at 6163 hg/ha, with Africa accounting for 6066 hg/ha, and Egypt accounting for 38748 hg/ha. The area under cowpea cultivation in Egypt is estimated to be 1853 ha. Cowpea is predominantly produced in more than 16 African countries (FAOSTAT, 2020). Because of its high nitrogen fixation ability, it is well adapted to growing in poor soils (Timko and Singh, 2008; EL-Ameen, 2018). It has a degree of tolerance to stress factors. However, salinity is one of the major abiotic stresses that severely affects cowpea crop production and quality (Hall, 2004; Eric et al., 2018). Yield is highly correlated with horticultural traits such as pod number per plant, pod length, and seed number per pod. As a result, any improved performance in these characteristics leads to an increase in yield (Ogunkanmi et al., 2013).

Cowpea genetic diversity has traditionally been assessed by measuring variability in phenotypic traits, which does not always have exceptionally sharp genetic relatedness (Patil et al., 2013; Eric et al., 2018). Furthermore, environmental factors have a strong impact on the interpretation of quantitative traits, limiting knowledge of the germplasm structure for the development of hybrids with specific environmental adaptive responses (Kumar, 1999; Animasaun et al., 2015; John and Clabe, 2016). Meanwhile, cross-breeding amongst cowpea genotypes is a very effective crop enhancement breeding approach. In the 1890s, the first crossings between crop cultivars and wild relatives to obtain disease-resistant varieties were made. Self-pollinating, hybridization to introduce inherited desired traits, and the use of lines and varieties as parents in crossover programs all contribute to cowpea’s inherent narrow genetic diversity (Nwosu and Awa, 2013; Patel et al., 2016). Prior research did not include wild relatives because breeders were concerned about their small seed size, seed coat color and texture, and pod shattering (Nkoana et al., 2019; Boukar et al., 2020). At the cellular level, three main processes govern plant growth and development: cell division (mitosis), cell expansion, and cell differentiation (Wang and Ruan, 2013). A cell population’s mitotic index has long been considered a crucial characteristic for cell and tissue development and multiplication. One reason for mitotic indexing of species is to generate data for breeding purposes (Darbelley et al., 1989; Driss-Ecole et al., 1994). The ongoing provision of new germplasm material as a donor of numerous agronomically significant genes is a critical condition for future development of cowpea cultivars, particularly given concerns that yield peaks in key crop species, including cowpea, have been achieved. Crossings among different cowpea varieties can surely help to the development of germplasm pools (Willie and Aikpokpodion, 2015). Better genotype development and selection is a crucial long-term method to fighting the problem of low yield in arid areas such as Egypt and other countries. One of the most efficient traditional breeding approaches is systematic germplasm development and evaluation of promising genotypes for adaptability and production stability. The goal of this study was to develop and evaluate new high-yielding cowpea crosses objectively. Meanwhile, the aim was to determine the genotypic and phenotypic variance and heritability of seeds yield and yield components in Egyptian developed cowpea crosses. The horticultural and cytological performance of five cowpea parental genotypes and six generated crosses were evaluated in F_1_ and F_2_ generations. The current study aimed to improve the characteristics of local commercial cultivars such Cream 7 cv. ‘Cr7’, Dokki 331 cv. ‘D331’, and Commercial 1 ‘Com1’ by crossing them with introduced cultivars such Colossus cv. ‘Col’, and Asian Introduction ‘AI’.

## 2 Materials and methods

### 2.1 Experimental site

The experiment was carried out at Minia University’s Horticulture Department, Faculty of Agriculture, El-Minia, Egypt. The field site is located at latitude 28°7’N and longitude 30°43’E. The research was carried out over three summer seasons in 2016, 2017, and 2018. Physical and chemical analyses of soil collected from a depth of 0.0 to 30 cm were performed over the seasons, and the average results are shown in **Supplementary Table 1**.

### 2.2 Parental genotypes

**Figure 1:**
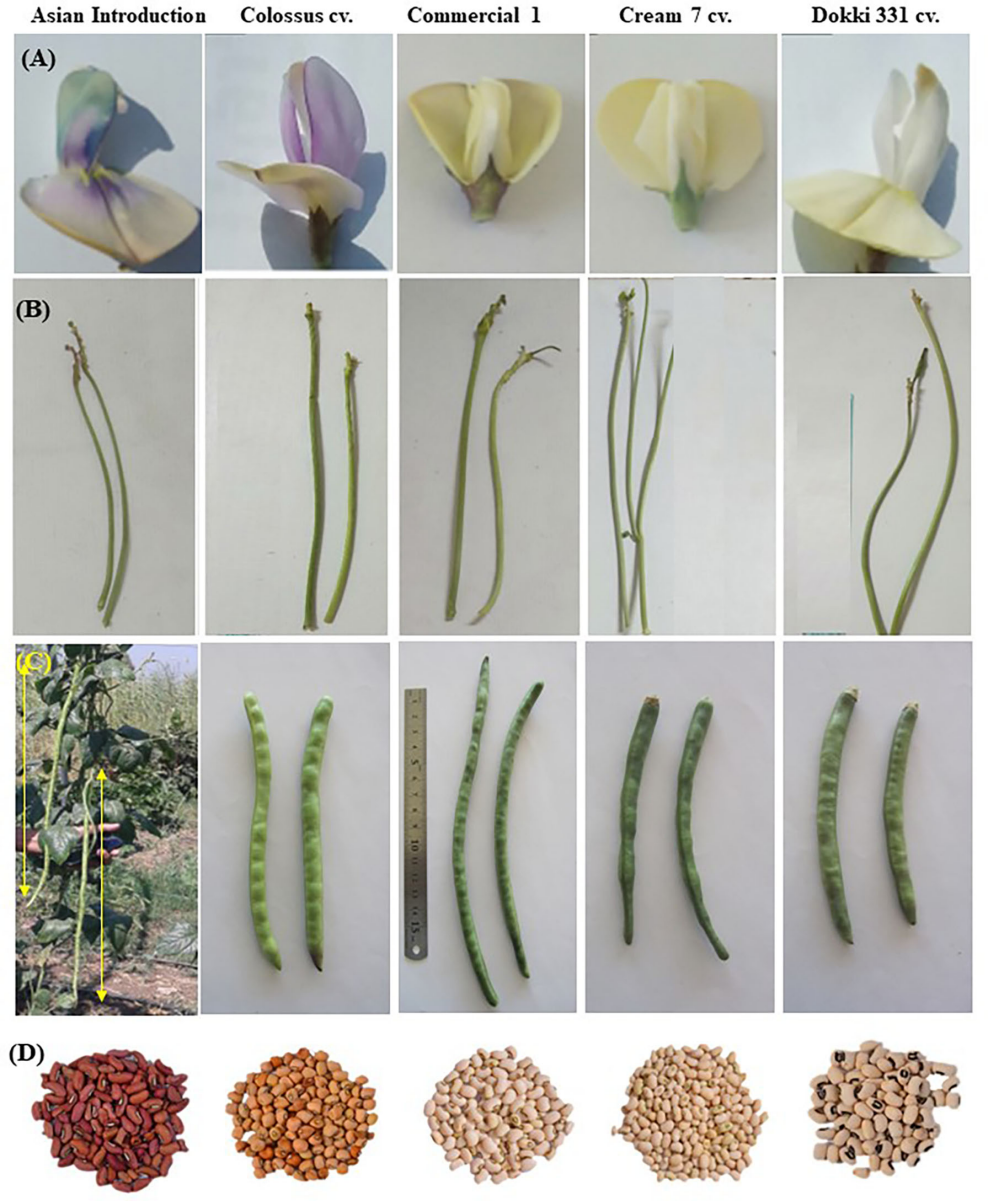
**(A)** flower traits, **(B)** peduncle length, **(C)** pod traits and **(D)** seed traits of five different cowpea parental genotypes examined under current investigation, i.e., and Asian Introduction, Colossus cv., Commercial 1, Cream 7 cv., and Dokki 331.

The present investigation searched for five different cowpea genotypes (*Vigna unguiculata* L. Walp). Local commercial cultivars, i.e., Cream 7 cv. ‘Cr7’, Dokki 331 cv. ‘D331’, and introduced cultivars, i.e., Colossus cv. ‘Col’ and Asian Introduction ‘AI’. Another cultivar, Commercial 1 ‘Com1’, was collected from the local market in El-Minia governorate for its seed quality characteristics. Twenty-eight cowpea varieties, including the current genotypes, were investigated for over 8 seasons in a comprehensive study undertaken by the author. Meanwhile, these genotypes were selected because of genetic variation in morphological, floral, pod, and seed traits (**Figure 1**), and genotypes such as ‘Cr7’ and ‘D331’ are commonly farmed in Egypt. All genotypes were selected for evaluation and cross experiments in the F_1_ and F_2_ generations. Genotype seeds were obtained from the Horticulture Department of the Faculty of Agriculture at Minia University in El-Minia, Egypt. The list of the examined genotypes with flower color, seed coat color, seed eye color, source and desirable traits is shown in **Table 1**.

**Table 1:** List of the tested parental genotypes with their flower color, seed color, source and desirable traits.

Parental genotypes*	Flower color	Seed coat color	Seed eye color	Source	Desirable traits
**Paternal parents**					
Asian Introduction ‘AI’	Purple	Cupreous	Colorless	Bangladesh	Pod length and number of seeds/pod
Commercial 1 ‘Com1’	White	White	Brown	Egyptian Market	Pod diameter, number of seeds/pod, seeds weight/pod
**Maternal parents**					
Colossus ‘Col’	Purple	Brown	Colorless	USA	Yield of seeds/plant
Cream 7 ‘Cr7’	White	Cream	Colorless	Egypt	number of pods/plant
Dokki 331 ‘D331’	White	White	Black	Egypt	Less number of aborted ovules/pod

^*^The study included five parental genotypes; local commercial cultivars, i.e., Cream 7 ‘Cr7’, Dokki 331 ‘D331’, and introduced cultivars, i.e., Colossus ‘Col’ and Asian Introduction ‘AI’. Another cultivar, Commercial 1 ‘Com1’, was collected from the local market for its seed’s quality characteristics.

### 2.3 Crossing experiment and evaluation

In this experiment, crossing among five cowpea genotypes, i.e., ‘Cr7’, ‘D331’, ‘Col’, ‘AI’, and ‘Com1’, was done to produce six crosses, which were studied in advanced generations of F_1_ and F_2_. Cultivars, ‘AI’ and ‘Com1’ were used as pollen donor parents, whereas the other cultivars, ‘Col’, ‘Cr7’ and ‘D331’ were employed as maternal parents. Crossing was carried out in the early morning by removing all anthers to prevent self-pollination and cutting all buds on the peduncle, followed by the application of pollen grains collected from donor plants to the pistil of the emasculated flowers. Wet cotton was used to cover the area of the removed buds, and paper bags were used to avoid cross-contamination with any foreign pollen grains. In 2016, through the crossing, F_1_ seeds of six crosses were obtained. In the meantime, F_1_ seeds were cultivated to produce F_2_ plants. The six crossings were evaluated for their morphological, yield, and yield component traits. Genotypes and crosses were distributed in plots, and each plot consisted of five rows (4 m length x 0.7 m wide), whereas the plot area was 14 m^2^. The inner three ridges were used for sampling, and the two outer ridges were left as guard ridges. The genotypes and crosses were organized using a Randomized Complete Block Design (RCBD). Each genotype was represented by a single plot, which was repeated three times. During the three seasons, seeds were sown at a rate of 2 seeds per hole, with a spacing of 25 cm between holes. During harvesting, crosses plants were sampled, and data was collected. All other agricultural practices were in accordance with commercial production guidelines.

### 2.4 Data collection and analysis

At harvesting time, plants and pods were chosen at random, and the morphological, floral, pods, seeds, and yield traits were examined.

#### 2.4.1 Morphological traits

Thirty plants were randomly selected from each plot of each genotype/cross, and the average shoot length, number of branches/plant, and stem diameter were investigated.

#### 2.4.2 Flower traits

The average length and diameter and number of peduncles/plant and flower length were examined in thirty plants which were randomly harvested from each genotype/cross.

#### 2.4.3 Pod and seed traits

The average number of pods/plant, seed weight, seed width, and seed length were calculated for thirty plants for each genotype/cross. Meanwhile, the average pod length, pod diameter, pod weight, number of seeds/pod, and number of aborted ovules/pod of ten pods were measured.

#### 2.4.4 Yield

The weight of the seeds per plant were measured in thirty plants randomly selected from each plot for each parental genotype and cross.

#### 2.4.5 Genetic analysis

The genotypic and phenotypic variance were estimated by using the following equations:

##### a. Genotypic variance


$$\sigma^{2}\mathrm{g=}\frac{MSt‐MSe}{r}$$

Whereas:

MSt = Mean sum of squares for trait of genotype/cross.

MSe = Mean sum of squares for error of genotype/cross.

r = Number of replications.

##### b. Phenotypic variance


$$\sigma^{2}\mathrm{p=}\sigma^{2}\mathrm{g+}\sigma^{2}e$$

Whereas:

σ^2^p = Phenotypic variance for each trait of genotype/cross.

σ^2^g = Genotypic variance for each trait of genotype/cross.

σ^2^e = Environmental variation among the tested traits of genotype/cross.

##### c. Phenotypic coefficient of variance and genotypic coefficient of variance

The PCV and GCV expressed as percentages were calculated as suggested by Burton and Vane (1953). In the meantime, PCV and GCV were classified into three classes; less than 10% (Low), 10 – 20% (Moderate) and more than 20% (High).


$$\text{Phenotypic Coefficient of Variance}\;(\text{PCV})=\frac{\sqrt{Phenotypic\;Variance}}{Mean}\times 100$$

$$\text{Genotypic Coefficient of Variance}\;(\text{GCV})=\frac{\sqrt{Genotypic\;Variance}}{Mean}\times 100$$

##### d. Heritability in broad sense

Heritability in broad sense meaning was estimated as the ratio of genetic variance to the phenotypic variance as reported by Burton (1952) as follow:


$$\text{Heritability }(\text{road sense})=\frac{Genotypic\;Variance}{Phenotypic\;Variance}\times 100$$

It was categorized according to Robinson et al. (1949) to three classes: 0.0-30% (Low), 31-60% (Medium) and more than 60% (High).

##### e. Genetic advance

Genetic advance as a percent of mean (GAM) was estimated and categorized as reported by Johnson et al. (1955) by the following formula:


$$GAM\%=\frac{K*H*p}{Mean}\times 100$$

Whereas:

K = 2.06 at 5% selection intensity.

H = Heritability.

P = Phenotypic standard deviation.

Meanwhile, GAM was categorized to three classes: less than 10% (Low), 10-20% (Moderate) and more than 20% (High).

#### 2.4.6 Cytological analysis

Cowpea seeds from the studied plant materials which included five parental genotypes and six crossings in F_1_ and F_2_ were germinated in Petri dishes with two layers of moist filter paper at room temperature for 48 hrs. Roots with a length of 1-2 cm were cut and fixed for 24 hrs. in a newly produced farmer’s fixative solution (absolute ethyl alcohol: glacial acetic acid, 3:1 v/v). The fixed roots were stored in 70% ethyl alcohol in the refrigerator at 4°C until analysis. The fixed roots were rinsed with distilled water, then hydrolyzed in 1 N HCl at 60°C for 10 min before being washed again. Mitotic investigations were conducted using the aceto-carmine squash preparation. For each genotype, almost 1000 cells were investigated (consisting of ten seeds). Images were captured with an Olympus BX51 microscope and a C-4040 zoom digital camera whenever possible. Mitotic index, phase index, and chromosomal aberrations were recorded for each genotype, and mitotic index was computed using Racuciu (2009) formula:


$$\mathrm{Mitotic index}=\frac{Total\;number\;of\;divided\;cells}{Total\;number\;of\;examined\;cells}\times 100$$

Percentage of abnormality of each stage of mitosis was counted for each slide.


$$\mathrm{Percentage of abnormality}\;=\frac{Total\;number\;of\;abnormal\;cells}{Total\;number\;of\;examined\;cells}\times 100$$

### 2.5. Statistical analysis

Data obtained from this study were subjected to analysis using SAS, version 9.3 (Cary, NC). Differences among cowpea genotypes were tested by an analysis of variance (ANOVA), and mean significant differences were tested by the Least Significant Difference (LSD) test at the 0.05 level of significance.

## 3 Results

### 3.1 Morphological traits

Morphological variability in shoot length, number of branches/plant, and stem diameter was noted among F_1_ and F_2_ generation crosses, as shown in **Table 2**. The shoot lengths of the parental genotypes ranged from 106.5 cm for ‘D331’ cv. with a range of 82-137 cm to 184.7 cm for ‘AI’ with a range of 167-210 cm. All six developed crossings produced F_1_ plants that were taller than F_2_ plants. F_1_ of Cr7 x AI cross had the shortest shoot length with a mean value of 122.7 cm, while D331 x AI cross had the largest shoot length with a mean value of 234.0 cm and exceeded the better parent. On the other hand, D331 x AI cross had the shortest shoot length of all F_2_ plants (133.3 cm), while Col x Com1 cross was superior and had the longest shoot (193.7 cm). Furthermore, the parental cultivar ‘D331’ had the highest number of branches per plant (7.4 branches) with a range of 4.0–12 branches, whilst ‘AI’ was the least branching with a mean value of 2.9 and a range of 2.0–4.0 branches. In comparison, F_1_ of Col x Com1 cross and F_2_ of Cr7 x AI cross produced the highest values of 9.0 and 5.5, with a range of 7.0–11 and 3–9 branches, respectively (**Table 2**). In the meantime, the mean stem diameter varied among the parental genotypes and crosses. ‘Cr7’ cv. produced a thicker diameter (1.9 cm) than other parental genotypes and crosses, with the exception of Col x Com1 cross in F_1_ plants, which had the largest stem diameter (2.0 cm). At the same time, the stem diameter of the obtained crosses ranged from 0.6 cm for cross Cr7 x AI with an average of 0.7 cm to 2.1 cm for cross Col x Com1 with an average of 2.0 cm in F_1_s, when it varied from 0.9 cm for Cr 7 x AI, Cr7 x Com 1, Col x AI, and D331 x AI to 2.4 cm for D331 x Com1 with an average of 1.6 cm in F_2_s. The data obtained, as given in **Table 2**, demonstrated that none of the developed crossings surpassed the diameter of the F_1_ cross’s Col x Com1 (2.0 cm).

**Table 2:** Mean and range of shoot length (cm), number of branches, stem diameter (cm), peduncle length (cm), peduncle diameter (mm), number of peduncles/plant, and flower length (cm) of six cowpea crosses produced from five different genotypes in F_1_, F_2_ generations.

Genotypes/Crosses	Shoot length (cm)		Number of branches/plant		Stem diameter (cm)		Peduncle length (cm)		Peduncle diameter (mm)		Number of Peduncles/ plant		Flower length (cm)	
	Mean	Range	Mean	Range	Mean	Range	Mean	Range	Mean	Range	Mean	Range	Mean	Range
Parental genotypes*														
Asian Introduction (AI)	184.7	167-210	2.9	2-4	1.1	1.0-1.3	24.4	17.3-31.3	0.3	0.3-0.4	18.2	10-23	2.7	2.5-2.9
Colossus (Col)	134.0	100-157	5.5	4-9	1.7	1.5-2.3	25.8	19.5-34.5	0.4	0.4-0.6	30.0	22-43	2.1	2.0-2.3
Commercial 1 (Com1)	139.2	115-190	4.1	3-6	1.3	1.0-1.8	29.1	21.0-40.5	0.4	0.3-0.6	27.8	20-62	2.3	2.0-2.5
Cream 7 (Cr7)	133.2	87-172	6.4	5-8	1.9	1.5-2.2	32.0	23.0-40.0	0.3	0.3-0.5	48.9	30-74	2.0	2.0-2.2
Dokki 331 (D331)	106.5	82-137	7.4	4-12	1.7	1.2-2.5	33.2	21.5-47.0	0.3	0.3-0.5	39.0	23-79	2.0	1.8-2.2
**Crosses (F_1_)**														
Col x AI	185.1	165-206	6.1	5-8	1.4	1.2-1.8	30.0	21.0-44.0	0.4	0.3-0.6	39.3	35-43	2.3	1.9-3.1
Col x Com1	220.5	189-252	9.0	7-11	2.0	1.9-2.1	42.0	41.0-43.0	1.1	0.6-1.7	55.0	48-62	2.0	1.7-2.1
Cr7 x AI	122.7	100-145	5.0	4-7	0.7	0.6-1.0	25.8	18.0-34.0	0.2	0.2-0.4	30.1	24-44	2.1	1.3-2.8
Cr7 x Com1	158.4	80-300	7.8	4-11	1.5	0.9-2.0	51.9	31.0-66.7	0.5	0.4-0.7	63.4	24-167	2.1	1.5-2.9
D331 x AI	234.0	233-235	8.5	6-11	1.3	1.0-1.6	24.4	10.1-30.0	0.4	0.4-0.5	49.4	32-69	1.9	1.7-2.2
D331 x Com1	146.4	125-212	6.8	5-9	1.6	1.2-1.9	37.5	35.5-39.5	0.3	0.3-0.4	41.5	33-50	2.1	1.8-2.9
**Crosses (F_2_)**														
Col x AI	183.0	125-264	5.0	3-8	1.2	0.9-2.1	37.2	23.2-49.2	0.3	0.3-0.5	28.3	15 -47	2.6	2.3-3.0
Col x Com1	193.7	120-271	5.0	3-8	1.4	1.1-1.8	36.6	23.1-51.8	0.5	0.4-0.7	29.8	19-41	2.5	2.2-3.1
Cr7 x AI	163.1	92-231	5.5	3-9	1.2	0.9-2.0	37.2	23.2-49.2	0.4	0.3-0.6	36.4	22-61	2.5	2.2-3.0
Cr7 x Com1	153.5	88-223	5.2	3-9	1.4	0.9-2.0	40.2	20.4-62.6	0.4	0.3-0.8	34.0	18-82	2.6	2.3-3.0
D331 x AI	133.3	86-198	4.8	3-7	1.3	0.9-2.0	34.4	23.4-58.8	0.4	0.3-0.8	27.8	17-41	2.5	2.0-3.0
D331 x Com1	157.4	105-289	5.3	3-8	1.6	1.1-2.4	35.5	19.4-50.7	0.4	0.3-0.6	34.2	22-51	2.5	2.3-3.0

*The study was conducted over three summer seasons in 2016, 2017, and 2018. F_1_ seeds from six crosses were obtained through crossing in 2016 meanwhile, F_1_ seeds were grown in 2017 to produce F_2_ seeds in 2018. The mean and range of the tested parents' scores, as well as statistics from the three growing seasons.

### 3.2 Flower traits

In terms of peduncle length, F_1_ and F_2_ of Cr7 x Com1 cross outperformed the other crosses, with a mean of 51.9 and 40.2 cm and a range of 31.0-66.7 cm and 20.4-62.6 cm, respectively. Nevertheless, this cross (Cr7 x Com1) produced a taller peduncle than the better parent, ‘D331’ cv., which had a peduncle length of 33.2 cm. The peduncle diameter of the parental genotypes, on the other hand, ranged from 0.3 to 0.4 cm, and better parents were ‘Col’ cv. and ‘Com1’ cv. The peduncle diameter of F_1_ plants ranged from 0.2 cm in Cr7 x AI cross to 1.1 cm in Col x Com1 cross. F_2_ of Col x Com1 cross produced the largest peduncle diameter, with an average of 0.5 cm and a range of 0.4 to 0.7 cm. These crosses outperformed the better parents, as indicated in **Table 2**.

The better parental genotype, ‘Cr7’ cv., produced the most peduncles (48.9) with a range of 30–74, whereas ‘AI’ produced the fewest peduncles (18.2) with a range of 10–23. In terms of the developed six crosses, the crosses in F_1_s had more peduncles than the crosses in F_2_s. F_1_ of Cr7 x Com1 cross produced the most peduncles (63.4) with a range of 24–167, outnumbering all other crosses and parental genotypes, while F_2_ of D331 x AI cross produced the fewest, still more than the least parent ‘AI’. Flower length also differed between parental genotypes and the six produced crosses in F_1_ and F_2_ generations. In general, when compared to other genotypes and crosses, ‘AI’ had the longest flower length. In contrast to the previously mentioned characteristics, F_2_ crosses had longer flower lengths than F_1_ crosses. The mean flower length in F_1_ and F_2_ crosses varied widely, from 2.3 cm for Col x AI cross to 2.6 cm for Cr7 x Com1 and Col x AI crosses. None of the tested crosses outperformed the flower length of the better genotype ‘AI’ (2.7 cm). On the other hand, F_1_ cross between the parental genotypes ‘D331’ and ‘Com1’ had the shortest flower length (1.9 cm) as shown in **Table 2**.

### 3.3 Pods and seed traits

**Figure 2:**
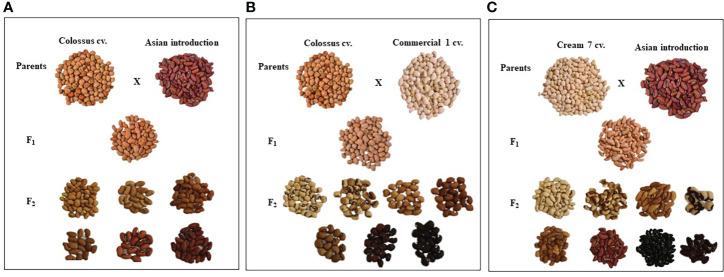
The diversification of seed characteristics of the parental genotypes, F_1_ and F_2_ of the crosses, **(A)** Colossus cv. x Asian Introduction, **(B)** Colossus cv. x Commercial 1and **(C)** Cream 7 cv. x Asian Introduction.

**Figure 3:**
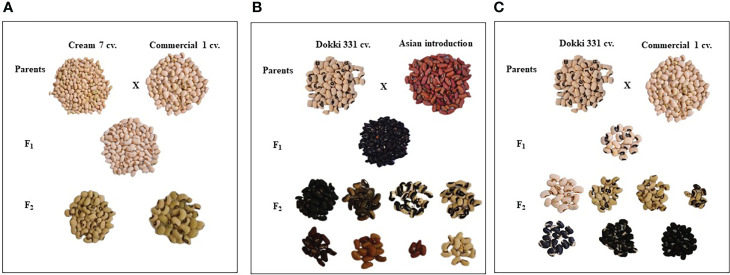
The diversification of seed characteristics of the parental genotypes, F_1_ and F_2_ of the crosses, **(A)** Cream 7 cv. x Commercial 1, **(B)** Dokki 331 cv. x Asian Introduction and **(C)** Dokki 331 cv. x Commercial 1.

Parental genotypes and all crosses in F_1_ and F_2_ generations showed considerable variations in pods and seed traits (**Figures 2**, **3**). The mean number of pods per plant of the developed crosses in this experiment differed from 21.5 for the cross Cr7 x AI to 71.0 for the cross D331 x AI in F_1_s, and from 38.7 for the cross Cr7 x Com1 to 48.2 for the cross Cr7 x AI in F_2_s. As reported in **Table 3**, the F_1_ of the crossing between ‘D331’ cv. and ‘AI’ outperformed the better parent, ‘Cr7’ cv. (58.9). The paternal parents, ‘AI’ and ‘Com1’ improved the pod traits in the six crosses. According to the approximated pod length values, F_1_ of the crossing between ‘D331’ cv. and ‘AI’ and F_2_ of the crossing between ‘Col’ and ‘AI’ showed the longest pod length (21.0 and 21.6 cm, respectively). However, none of the crosses exceeded the better parent, ‘AI’, which had the tallest pod with an average of 34.2 cm and a range of 24.0 to 42.0 cm (**Table 3**). All produced crossings in F_1_s and F_2_s had thicker pods than the parental genotypes in terms of pod diameter, which was particularly noticeable in F_1_ crosses. Pod diameter ranged from 0.3 to 0.5 cm in the parental genotypes, while it ranged from 0.6 to 0.8 cm in F_1_ crossings and from 0.6 to 0.7 cm in F_2_ crosses. The maximum pod diameter was found in F_1_ crosses of Cr7 x AI, Col x Com1, and Col x AI (**Table 3**).

**Table 3:** Mean and range of number of pods/plant, pod length (cm), pod diameter (cm), fresh pod weight (g), and number of aborted ovules/pod of six cowpea crosses produced from five different genotypes in F_1_, F_2_ generations.

Genotypes/Crosses	Number of pods/plant		Pod length(cm)		Pod diameter (cm)		Fresh pod weight (g)		Number of aborted ovules /pod	
	Mean	Range	Mean	Range	Mean	Range	Mean	Range	Mean	Range
Parental genotypes*										
Asian Introduction (AI)	18.3	10.0-27.0	34.2	24.0-42.0	0.3	0.3-0.5	2.3	0.7-3.4	8.6	3.0-13.0
Colossus (Col)	27.0	15.0-42.0	13.2	11.0-15.1	0.5	0.5-0.6	3.3	1.5-4.5	5.6	3.0-11.0
Commercial 1 (Com1)	30.1	16.0-90.0	19.4	14.7-23.5	0.5	0.5-0.6	3.7	2.5-4.7	5.1	2.0-9.0
Cream 7 (Cr7)	58.9	21.0-94.0	13.6	9.7-17.0	0.4	0.4-0.5	1.6	0.7-2.7	6.3	3.0-10.0
Dokki 331 (D331)	35.3	17.0-74.0	13.7	10.1-16.0	0.4	0.4-0.5	2.2	1.1-2.9	4.1	2.0-8.0
**Crosses (F_1_)**										
Col x AI	41.1	37.0-55.0	19.4	8.6-29.5	0.8	0.6-0.9	2.1	0.6-3.7	7.3	3.0-12.0
Col x Com1	30.0	22.0-38.0	16.1	10.2-21.5	0.8	0.7-1.0	2.7	0.6-4.7	5.2	2.0-13.0
Cr7 x AI	21.5	14.0-34.0	19.6	13.3-29.0	0.8	0.7-1.0	1.6	0.9-2.7	7.8	5.0-12.0
Cr7 x Com1	55.5	33.0-88.0	17.6	12.4-20.3	0.7	0.6-0.8	2.4	1.0-3.2	4.5	2.0-7.0
D331 x AI	71.0	55.0-87.0	21.0	16.6-25.8	0.7	0.7-0.8	2.6	1.8-3.6	4.6	2.0-8.0
D331 x Com1	49.8	42.0-58.0	13.3	10.1-15.7	0.6	0.6-0.8	1.7	1.0-2.7	4.8	2.0-10.0
**Crosses (F_2_)**										
Col x AI	40.3	23.0-96.0	21.6	11.5-44.3	0.6	0.5-0.9	2.3	0.6-4.1	5.7	2.0-11.0
Col x Com1	38.8	28.0-53.0	18.0	11.2-22.8	0.7	0.6-0.9	3.0	1.6-5.4	4.1	0.0-11.0
Cr7 x AI	48.2	23.0-69.0	19.1	7.4-37.3	0.6	0.5-1.0	1.5	0.3-3.2	5.6	2.0-13.0
Cr7 x Com1	38.7	15.0-74.0	15.5	9.4-20.5	0.6	0.5-1.1	1.9	0.4-3.2	4.7	1.0-14.0
D331 x AI	40.8	28.0-61.0	20.9	13.2-26.3	0.7	0.5-0.9	2.8	1.5-4.2	5.1	0.0-9.0
D331 x Com1	39.7	27.0-58.0	13.8	8.9-22.7	0.6	0.4-1.0	1.3	0.6-2.7	5.3	2.0-9.0

*The study was conducted over three summer seasons in 2016, 2017, and 2018. F^1^ seeds from six crosses were obtained through crossing in 2016 meanwhile, F_1_ seeds were grown in 2017 to produce F_2_ seeds in 2018. The mean and range of the tested parents' scores, as well as statistics from the three growing seasons.

When it came to fresh pod weight, there were substantial differences among the tested genotypes and the six developed crosses in F_1_ and F_2_. The best parent was ‘Com1’ with an average of 3.7 g per pod, followed by ‘Col’ cv. with an average of 3.3 g per pod, whilst ‘D331’ cv. and ‘Cr7’ cv. were the least parents with an average of 2.2 and 1.6 g per pod, respectively. Meanwhile, ‘Com1’ cv. had the maximum average pod weight, followed by ‘Col’ cv., with no significant differences however, ‘Cr7’ cv. had the lowest pod weight values across the three seasons (**Table 3**). In F_1_ crossings, pod weights varied from 1.6 g for Cr7 x AI cross to 2.7 g for Col x Com 1 cross, whereas, in F_2_ crossings, pod weights ranged from 1.3 g for D331 x Com 1 cross to 3.0 g for Col x Com 1 cross. 'Com 1' was the better parent, with the greatest pod weight of 3.7 g on average and a range of 2.5–4.7 g. Meanwhile, as seen in **Table 3**, none of the crosses outperformed the superior parent.

In parental genotypes, the number of aborted ovules ranged from 4.1 to 8.6 ovules/pod, while in F_1_ and F_2_ crossings, the number ranged from 4.1 to 7.8 ovules/pod. ‘D331’ cv. yielded the greatest results and had the fewest aborted ovules/pod (4.1 ovules/pod), while the F_2_ cross of Col x Com1 yielded the same result. The largest number of aborted ovules were found in ‘AI’ and F_1_ of Cr7 x AI cross, with 8.6 and 7.8, respectively (**Table 3**).

**Table 4** reveals that there were significant differences in seed length, seed width, and seed weight/pod between genotypes and crosses in F_1_ and F_2_.The parental genotype ‘AI’ and F_1_ of D331 x AI cross had the greatest value (1.0 cm in seed length). On the other hand, ‘Cr7’ cv. and F_2_ of Cr7 x Com1 cross had the lowest value (0.7 cm in seed length). At the same time in this trial, the mean seed width of the six crossings produced increased from 0.4 to 0.5 in F_1_s and F_2_s. In comparison to the other crosses, F_1_ and F_2_ of Col x Com1 cross generated the greatest values of 0.5 cm, but the crosses did not surpass the better parent, ‘Col’ cv. which had 0.6 cm in seed width (**Table 4**). Crossing among the parents improved the number of seeds/pod trait and it was considerably clear in the crosses in F_2_s (**Table 4**). The paternal genotype, ‘AI’ was the better parent for the number of seeds per pod, with an average of 11.0 seeds and a range of 3.0-15.0 seeds. Four of the developed crosses, D331 x AI and Cr7 x Com1 in F_1_ and Col x Com1 and D331 x AI in F_2_ crosses, were higher than the better parent. Nevertheless, F_2_ of Col x Com1 cross, followed by F_1_ and F_2_ of D331 x AI cross, produced the highest number of seeds/pod (11.9 and 11.7 seeds/pod, respectively). **Figures 2**, **3** illustrated the seed traits of the six produced crosses. Parental genotypes and developed crosses had a considerable variation in seed color, seed coat color and size. Furthermore, the seed weight/pod of the parental genotypes varied from 1.3 g for ‘AI’ to 2.7 g for ‘Com1’. For crosses between parental genotypes, they ranged from 0.9 g for F_1_ of Cr7 x AI cross to 2.0 g for F_1_ of Col x Com1 cross. However, in F_2_, the seed weight ranged from 0.9 g for D331 x Com1 cross to 2.2 g for Col x Com1 cross. Meanwhile, Col x Com1 cross was superior in the two generations but still weighted less than the better parents, ‘Com1’ cv. (2.7 g) and ‘Col’ cv. (2.6 g) as clear in **Table 4**.

**Table 4:** Mean and range of seed length (mm), seed width (mm), number of seeds/pod, seeds weight/pod (g) and seeds weight/plant (g) of six cowpea crosses of F_1_, F_2_ generations which produced from five different genotypes.

Genotypes/Crosses	Seed length (mm)		Seed width (mm)		Number of seeds/pod		Seeds weight/pod (g)		Seeds weight/plant (g)	
	Mean	Range	Mean	Range	Mean	Range	Mean	Range	Mean	Range
Parental genotypes*										
Asian Introduction (AI)	1.0	0.9-1.1	0.3	0.3-0.4	11.0	3.0-15.0	1.3	0.1-2.3	13.3	10.1-18.5
Colossus (Col)	0.8	0.7-0.9	0.6	0.6-0.7	10.4	5.0-15.0	2.6	1.0-4.0	23.1	17.2-27.2
Commercial 1 (Com1)	0.9	0.8-1.2	0.5	0.5-0.6	10.3	6.0-16.0	2.7	1.7-3.7	24.7	18.6-31.6
Cream 7 (Cr7)	0.7	0.7-0.8	0.4	0.4-0.6	8.1	3.0-11.0	1.0	0.3-1.9	16.2	11.8-21.4
Dokki 331 (D331)	0.9	0.8-1.2	0.5	0.4-0.6	8.6	4.0-12.0	1.8	0.8-2.4	17.7	12.0-23.0
**Crosses (F_1_)**										
Col x AI	0.9	0.8-1.2	0.4	0.4-0.6	7.7	2.0-12.0	1.3	0.3-2.6	43.6	38.1-55.9
Col x Com1	0.8	0.8-1.0	0.5	0.4-0.6	9.9	3.0-16.0	2.0	0.7-2.1	46.1	27.9-46.3
Cr7 x AI	0.9	0.8-1.1	0.4	0.3-0.5	7.6	5.0-12.0	0.9	0.5-1.4	15.4	8.3-26.6
Cr7 x Com1	0.8	0.8-1.0	0.4	0.4-0.5	11.3	5.0-15.0	1.7	0.7-2.5	67.2	35.5-106.6
D331 x AI	1.0	0.8-1.1	0.4	0.4-0.5	11.7	7.0-15.0	1.8	0.8-2.8	80.7	55.4-106.1
D331 x Com1	0.8	0.7-1.1	0.4	0.4-0.5	7.5	4.0-10.0	1.3	2.1-0.7	53.7	47.7-61.9
**Crosses (F_2_)**										
Col x AI	0.9	0.8-1.3	0.4	0.4-0.6	11.0	3.0-18.0	1.7	0.3-3.1	27.7	10.2-59.0
Col x Com1	0.8	0.7-0.9	0.5	0.4-0.6	11.9	6.0-20.0	2.2	0.9-4.4	22.9	11.1-51.7
Cr7 x AI	0.8	0.7-1.2	0.4	0.3-0.5	8.8	3.0-16.0	1.0	0.1-2.4	23.9	11.7-42.5
Cr7 x Com1	0.7	0.6-1.0	0.4	0.4-0.5	10.0	2.0-16.0	1.4	0.2-2.6	17.5	5.0-39.0
D331 x AI	0.9	0.9-1.1	0.4	0.4-0.5	11.7	7.0-17.0	2.1	0.9-3.4	23.1	12.1-40.1
D331 x Com1	0.8	0.6-1.1	0.4	0.3-0.7	7.2	2.0-13.0	0.9	0.2-2.0	19.3	7.6-33.8

*The study was conducted over three summer seasons in 2016, 2017, and 2018. F^1^ seeds from six crosses were obtained through crossing in 2016 meanwhile, F_1_ seeds were grown in 2017 to produce F_2_ seeds in 2018. The mean and range of the tested parents' scores, as well as statistics from the three growing seasons.

### 3.4 Seed yield

The seed weight per plant produced by the F_1_ and F_2_ generations of crossings, as well as the parental genotypes, indicated significant variation. In parental genotypes, ‘Com1’ and ‘Col’ cv. yielded higher seed weight than the others, with mean values of 24.7 and 23.1 g/plant, respectively, while ‘AI’ produced the least of all genotypes and examined crosses. Crosses in F_1_ had a higher seed weight than crosses in F_2_, with the exception of Cr7 x AI cross, which had the lowest seed weight of all the crosses. D331 x AI cross exceeded the parental genotypes as well as the other crosses in F_1_ with an average of 80.7 g/plant and a range of 55.4-106.1 g/plant. Col x AI had heavier seeds in F_2_s than other crosses with an average of 27.7 g/plant and a range from 10.2-59.0 g/plant, which was still greater than the better parents (**Table 4**).

### 3.5 Genetic parameters analysis

**Tables 5**, **6** present the findings of the genetic investigation among the parental genotypes, as well as the crosses seen in F_1_ and F_2_. The successful crosses were accomplished using ‘Cr7’ cv., ‘Col’ cv. and ‘D331’ cv. as female parents and ‘AI’ and ‘Com1’ genotypes as male parents. Meanwhile, phenotypic variance (PV), genotypic variance (GV), phenotypic coefficient of variance (PCV %), genotypic coefficient of variance (GCV %), heritability (H %), and genetic advance mean (GAM) of number of pods/plant, pod length, number of seeds/pod, number of aborted ovules/pod, fresh pod weight, seeds weight/pod and seeds yield/plant of the five parental genotypes and six crosses in F_1_ and F_2_ were evaluated. Other morphological and floral parameters, such as shoot length, number of branches/plant, peduncle length, and number of peduncles/plant, were also genetically analyzed for parental genotypes and crosses as clear in **Supplementary Tables 2**, **3**, respectively.

**Table 5:** Genotypic variance (GV), phenotypic variance (PV), genotypic coefficient of variance (GCV %), phenotypic coefficient of variance (PCV %), heritability (H%), and genetic advance mean (GAM) of pods, seeds traits and yield of the five parental genotypes; local commercial cultivars, i.e., Cream 7 ‘Cr7’, Dokki 331 ‘D331’, and introduced cultivars, i.e., Colossus ‘Col’ and Asian Introduction ‘AI’. Another cultivar, Commercial 1 ‘Com1’, was collected from the local market in El-Minia governorate for its seed’s quality characteristics.

Parental genotypes	Traits*	GV	PV	GCV %	PCV %	H %	GAM
AI	NPo/P	32.011	30.22	30.91	30.04	94.41	60.13
	PoL	26.3	24.7	14.98	14.51	93.84	28.97
	NS/Po	19.5	18.1	40.20	38.73	92.85	76.89
	NAO/Po	13.6	12.4	42.88	41.00	91.42	80.76
	FPoW	0.62	0.37	33.70	26.13	60.12	41.76
	SW/Po	0.57	0.33	55.64	42.49	58.33	66.86
	SY/P	103.91	100.69	32.83	32.31	96.89	65.53
Col cv.	NPo/P	498.32	491.26	74.16	73.63	98.58	150.61
	PoL	5.44	4.71	12.01	11.17	86.45	21.39
	NS/Po	8.23	7.32	27.85	26.27	88.98	51.05
	NAO/Po	5.21	4.48	44.76	41.54	86.14	79.43
	FPoW	0.69	0.42	22.13	17.42	61.99	28.27
	SW/Po	0.50	0.27	25.48	18.95	55.35	29.06
	SY/P	123.06	119.55	35.13	34.62	97.14	70.30
Com1	NPo/P	66.44	63.86	30.19	29.59	96.12	59.77
	PoL	1.84	1.41	10.27	8.99	76.68	16.22
	NS/Po	11.8	10.7	33.06	31.50	90.80	61.83
	NAO/Po	5.37	4.64	41.41	38.48	86.37	73.67
	FPoW	1.35	0.98	34.94	29.80	72.76	52.36
	SW/Po	1.14	0.80	39.87	33.45	70.40	57.84
	SY/P	1206.4	1195.4	71.13	70.81	99.08	145.2
Cr7 cv.	NPo/P	275.12	269.87	46.98	46.53	98.09	94.95
	PoL	4.34	3.68	15.13	13.93	84.82	26.44
	NS/Po	8.71	7.77	34.31	32.42	89.28	63.11
	NAO/Po	3.65	3.05	46.63	42.60	83.45	80.16
	FPoW	0.38	0.18	26.93	18.79	48.69	27.00
	SW/Po	0.27	0.10	28.92	18.08	39.11	23.27
	SY/P	131.93	128.30	41.25	40.68	97.24	82.64
D331 cv.	NPo/P	744.3	735.6	46.31	46.05	98.84	94.31
	PoL	3.62	3.02	13.99	12.77	83.37	24.03
	NS/Po	7.43	6.57	33.65	31.64	88.40	61.28
	NAO/Po	6.90	6.06	41.69	39.10	87.95	75.55
	FPoW	0.30	0.13	33.83	22.09	42.62	29.69
	SW/Po	0.19	0.05	41.38	22.34	29.14	24.84
	SY/P	216.02	211.38	52.57	52.00	97.84	105.9

*Number of pods/plant (NPo/P), pod length (PoL), number of seeds/pod (NS/Po), number of aborted ovules /pod (NAO/Po), fresh pod weight (FPoW), seeds weight/pod (SW/Po) and seeds yield/plant (SY/P).

**Table 6:** Genotypic variance (GV), phenotypic variance (PV), genotypic coefficient of variance (GCV %), phenotypic coefficient of variance (PCV %), heritability (H%), and genetic advance mean (GAM) of pods, seeds traits and yield of the six crosses in F_1_ and F_2_ produced from crossing between five parental genotypes; Cream 7 cv. (Cr7), Colossus (Col) cv., and Dokki 331 cv. (D331) genotypes as female parents and Asian Introduction (AI) and Commercial 1 (Com1) genotypes as male parents.

Crosses	Traits*	GV		PV		GCV %		PCV %		H %		GAM	
		F_1_	F_2_	F_1_	F_2_	F_1_	F_2_	F_1_	F_2_	F_1_	F_2_	F_1_	F_2_
Col x AI	NPo/P	57.2	92.54	53.81	91.02	18.37	23.29	17.82	23.1	94.08	98.35	35.61	47.19
	PoL	21.1	49.4	20.1	48.3	23.71	29.18	23.13	28.85	95.13	97.75	46.47	58.76
	NS/Po	7.90	18.7	7.27	18.0	36.51	35.19	35.03	34.54	92.04	96.34	69.23	69.85
	NAO/Po	7.27	5.60	6.67	5.22	36.94	46.40	35.38	44.82	91.71	93.32	69.79	89.18
	FPoW	0.90	1.09	0.69	0.93	45.15	38.79	39.49	35.73	76.49	84.86	71.14	67.79
	SW/Po	0.55	0.68	0.38	0.55	55.44	44.29	46.36	39.83	69.92	80.90	79.86	73.79
	SY/P	52.30	175.71	49.07	173.6	16.55	43.82	16.03	43.55	93.81	98.80	31.99	89.19
Col x Com1	NPo/P	128.0	54.64	120.0	53.47	37.71	19.25	36.51	19.04	93.75	97.86	72.83	38.80
	PoL	14.3	8.98	13.4	8.51	23.52	16.56	22.81	16.11	94.09	94.72	45.59	32.32
	NS/Po	14.3	13.5	13.4	12.9	38.20	30.90	37.05	30.23	94.08	95.69	74.04	60.93
	NAO/Po	7.03	5.68	6.44	5.30	51.02	59.59	48.82	57.58	91.57	93.36	96.24	114.6
	FPoW	1.29	0.94	1.03	0.79	40.87	32.51	36.63	29.74	80.32	83.70	67.62	56.05
	SW/Po	0.90	0.74	0.69	0.6	46.18	39.87	40.38	36.03	76,46	81.67	72.74	67.06
	SY/P	662.8	74.1	644.63	72.74	55.79	40.20	55.02	39.83	97.25	98.16	111.7	81.31
Cr7 x AI	NPo/P	33.35	274.5	31.68	271.9	26.86	32.48	26.18	32.33	95.00	99.04	52.57	66.29
	PoL	15.5	49.2	14.6	48.1	20.09	31.75	19.51	31.39	94.31	97.74	39.03	63.95
	NS/Po	5.18	9.85	4.67	9.36	29.96	36.93	28.45	35.99	90.18	94.96	55.67	72.24
	NAO/Po	3.29	6.02	2.88	5.64	23.26	40.91	21.77	39.58	87.66	93.56	42.00	78.86
	FPoW	0.26	0.53	0.15	0.42	31.24	44.37	23.56	39.27	56.87	78.35	36.60	71.61
	SW/Po	0.10	0.32	0.03	0.23	33.89	51.82	19.20	44.06	32.11	72.30	22.42	77.18
	SY/P	43.44	58.13	41.54	56.93	42.71	30.06	41.77	29.75	95.61	97.92	84.14	60.65
Cr7 x Com1	NPo/P	427.3	150.0	420.8	148.0	37.24	30.77	36.96	30.57	98.47	98.70	75.56	62.57
	PoL	4.81	6.79	4.32	6.37	12.46	16.18	11.81	15.68	89.81	93.93	23.05	31.32
	NS/Po	8.01	12.9	7.37	12.3	25.04	34.53	24.03	33.76	92.09	95.59	47.52	68.01
	NAO/Po	2.47	8.24	2.11	7.78	34.93	62.40	32.34	60.65	85.75	94.49	61.70	121.4
	FPoW	0.36	0.42	0.22	0.31	24.92	31.80	19.75	27.64	62.77	75.53	32.23	49.50
	SW/Po	0.29	0.31	0.17	0.22	30.94	37.42	23.74	31.69	58.84	71.70	37.51	55.21
	SY/P	550.5	77.17	543.08	75.78	34.89	49.35	34.65	48.90	98.65	98.2	70.91	43.01
D331 x AI	NPo/P	512.0	74.98	496.0	73.61	31.86	20.71	31.36	20.52	96.87	98.17	63.59	41.89
	PoL	6.31	8.61	5.75	8.14	11.96	14.38	11.42	13.99	91.10	94.61	22.45	28.03
	NS/Po	6.82	42.3	6.24	41.2	22.33	26.43	21.35	26.11	91.44	97.56	42.07	42.80
	NAO/Po	2.34	4.91	2.00	4.56	33.28	41.04	30.76	39.55	85.41	92.87	58.57	78.51
	FPoW	0.34	0.60	0.21	0.47	21.70	29.47	17.03	26.28	61.58	79.53	27.53	48.27
	SW/Po	0.24	0.5	0.13	0.39	26.43	35.63	19.61	31.42	55.06	77.75	29.98	57.02
	SY/P	1284.2	42.30	1258.8	41.27	44.36	26.43	43.92	26.11	98.02	97.56	89.58	53.14
D331 x Com1	NPo/P	54.20	70.56	50.90	69.23	14.78	21.59	14.32	21.39	93.92	98.11	28.60	43.64
	PoL	2.71	6.82	2.34	6.4	12.37	19.2	11.50	18.61	86.42	93.94	22.03	37.16
	NS/Po	4.68	5.83	4.20	5.45	28.85	34.01	27.32	32.88	89.66	93.45	53.30	65.48
	NAO/Po	6.23	2.94	5.68	2.67	52.03	31.76	49.69	30.27	91.04	90.79	97.59	59.43
	FPoW	0.16	0.21	0.07	0.14	23.82	32.80	16.01	26.63	45.18	65.89	22.17	44.54
	SW/Po	0.12	0.14	0.04	0.08	26.75	38.21	15.95	29.37	35.53	59.06	19.58	46.49
	SY/P	44.89	46.84	41.90	45.76	12.46	35.17	12.04	34.76	93.32	97.69	23.96	70.78

*Number of pods/plant (NPo/P), pod length (PoL), number of seeds/pod (NS/Po), number of aborted ovules /pod (NAO/Po), fresh pod weight (FPoW), seeds weight/pod (SW/Po) and seeds yield/plant (SY/P).

The genetic analysis revealed significant differences between the parental genotypes (**Table 5**) and obtained crosses (**Table 6**). For the parental genotypes, high and moderate genotypic and phenotypic coefficients of variance (PCV %) were obtained for the number of pods/plant. In F_1_ and F_2_, the genotypic variance (GV) was greater than the phenotypic variance (PV) in the five parents and the six crossings. Simultaneously, GCV % had greater impacts than PCV % in the studied characteristics. All parents and six crossings in F_1_ and F_2_ had high heritability values. GAM ranges were found to be broad for all parents and crosses in F_1_ and F_2_. Heritability values for the parental genotypes and F_1_ and F_2_ crossings ranged from 76.68% to 96.96%, with a high H% for pod length. With the exception of ‘D331’ cv., which showed low values for PCV %, GCV %, and GAM, all parental genotypes had moderate PCV % and GCV %, whereas the six crossings in F_1_ and F_2_ had high GAM values for the trait, as shown in **Tables 5**, **6**.

The H % and GAM % of the number of seeds/pod were both high in all parents and the resulting F_1_ and F_2_ crosses (**Tables 5** and **6**). The estimations of PCV % were lower in all genotypes than the estimations of GCV %, indicating that this trait is influenced by the environment. Furthermore, the PCV %, GCV %, H %, and GAM of the number of aborted ovules per pod were all high in the five parents (**Table 5**). When compared to the other parental genotypes, ‘AI’ and ‘Col’ cv. demonstrated greater values. All F_1_ and F_2_ crossings yielded high values for the genetic characteristics tested for the number of aborted ovules/pod. F_1_ of Col x AI cross and F_2_ of Cr7 x Com1 cross had the highest H % with 91.71% and 94.49%, respectively (**Table 6**).

PCV %, GCV %, H %, and GAM of fresh pod weight were all high for all parental genotypes excluding the ‘D331’ cv., which had low values for PCV %, H %, and GAM, 8.44%, 18.07%, and 6.70%, respectively. All crosses in F_1_ and F_2_ had high H % values, other than F_1_ crossings D331 x Com1 and Cr7 x AI, which had moderate H % values of 45.18% and 56.87%, respectively. GAM of all F_1_ and F_2_ crosses revealed elevated fresh pod weight values. At the same time, all F_1_ and F_2_ crosses showed high PCV % and GCV %, apart from D331 x Com1 and Cr7 x Com1, which had moderate PCV % values of 16.01% and 19.75%, respectively (**Tables 5**, **6**).

The H % of seeds weight per pod was low, medium, and high for the parental genotypes studied. The GAM of seeds weight/pod values was high in ‘D331’ cv., ‘Cr7’ cv., ‘Com1’, ‘Col’ cv., and ‘AI’. The PCV and GCV % of the parents examined suggested moderate and high levels of the analyzed trait. In F_1_ crossings, seeds weight heritability varied from 32.11% to 76.46%. Similarly, the proportion of F_2_ crossings varied between 59.06 and 81.67%. Meanwhile, F_1_ and F_2_ crossings produced moderate and high PCV % and GCV % of seeds weight/pod values, respectively. Seeds weight per plant of the five parents and six crossings in F_1_ and F_2_ showed greater GCV % values than PCV %, indicating that genotypes interact with environmental variables to influence the expression of this trait which was found to have a high H % and GAM. In general, genetic diversity and the heritability of desired trait control the overall performance of crop development and breeding (**Tables 5**, **6**).

### 3.6 Cytological analysis of parental genotypes and crossings

The mean proportions of phase and mitotic index were measured in root meristem cells from the five parents and six F_1_ and F_2_ crossings (**Table 7**). The mean percentages of mitotic index (MI) ranged from 2.56 to 4.53% in parental genotypes, from 2.63% to 4.53% in F_1_, and from 2.39% to 4.31% in F_2_. The maximum amount of MI (4.53%) was obtained from Col x AI cross in F_1_ and ‘AI’ genotype, whereas the lowest value (2.39%) was obtained from Cr7 x Com1 cross. Except for the Col x AI cross in F_1_ and the ‘AI’ genotype, which had the same value (4.53%), and Col x AI cross in F_2_ (4.31%), the percentages of MI were almost same with in all genotypes/crosses. On the other hand, prophase index data revealed a significant difference across all studied parents and crossings. Furthermore, ‘Col’ cv. exhibited the greatest percentage of prophase index (44.60%), with a substantial rise above almost all other genotypes. ‘Com 1’ cv., on the other hand, had the lowest proportion (18.34%). Nevertheless, as shown in **Table 7**, Cr7 x AI cross, followed by Col x AI cross in F_1_, exhibited the greatest prophase index of 36.84% and 33.46%, respectively, when compared to the other produced crossings in F_1_ and F_2_. Metaphase index values were often greater than previous phases. Among all genotypes, D331 x AI F_1_ cross had the highest metaphase index (66.23%), whereas ‘Col’ cv. produced the lowest (39.84%). Despite the fact that Col x AI cross in F_2_ (26.22%), which was greater than its F_1_ value (19.16%), the anaphase and telophase indexes appeared at a low frequency compared to other phases.

**Table 7:** Proportions of phase and mitotic index (MI) derived from the root tips of six crosses in F_1_ and F_2_ produced from crossing between five parental genotypes; Cream 7 cv. ‘Cr7’, Colossus cv. ‘Col’, and Dokki 331 cv. ‘D331’ genotypes as female parents and Asian Introduction ‘AI’ and Commercial 1 ‘Com1’ genotypes as male parents.

Genotypes	Number of examined cells	Prophase %	Metaphase %	Ana & telophase %	Mitotic index %
**Parental parents**					
Asian Introduction (AI)	1165	27.80	52.11	20.09	4.53
Colossus (Col)	1108	44.60	39.84	15.56	2.56
Commercial 1 (Com1)	1153	18.34	57.04	24.61	2.63
Cream 7 (Cr7)	1046	19.22	55.07	25.70	2.75
Dokki 331 (D331)	1171	18.46	61.33	20.21	2.96
**Crosses (F_1_)**					
Col x AI	1172	33.46	47.38	19.16	4.53
Col x Com1	1234	24.97	54.19	20.84	3.80
Cr7 x AI	1151	36.84	40.24	22.92	3.41
Cr7 x Com1	1220	30.22	50.91	18.86	2.63
D331 x AI	1182	18.53	66.23	15.24	2.75
D331 x Com1	1210	23.79	60.66	15.55	3.33
**Crosses (F_2_)**					
Col x AI	1364	29.94	43.84	26.22	4.31
Col x Com1	1267	26.63	54.09	19.28	3.53
Cr7 x AI	1359	25.46	55.92	18.62	2.84
Cr7 x Com1	1455	23.25	60.63	16.13	2.39
D331 x AI	1347	25.64	53.57	20.80	2.51
D331 x Com1	1383	29.80	50.34	19.87	2.75
LSD_0.05_		11.83	10.77	11.39	1.23

**Table 8** demonstrated many forms of mitotic abnormalities such as lagging chromosomes, chromosomal bridges, outside chromosomes, stickiness, and micronuclei. When compared to all other crosses and parental genotypes, D331 x Com1 cross in F_1_ and ‘Cr7’ cv. provided the highest values of total mitotic abnormalities (6.61% and 5.77%, respectively), while the two crosses in F_2_, Col x AI and D331 x AI recorded the lowest values (0.93% and 1.01%, respectively). The percentage of remained genotypes with abnormalities varied from 1.43% to 5.57%. **Table 8** also showed that the two genotypes, ‘Col’ cv. and Cr7 x Com1 cross in F_2_, had the greatest frequencies of chromosomal bridges (2.06% and 1.96%, respectively) compared to the other parental genotypes and crosses studied. Almost all cowpea genotypes tested positive for the outside chromosome. It was found in high frequency in D331 x Com1 cross in F_1_ and ‘Cr7’ cv. (2.66% and 2.16%, respectively) with considerable differences with all other genotypes. In terms of chromosomal stickiness %, the aforementioned genotypes ‘Cr7’ cv. followed by D331 x Com1 cross in F_1_ displayed the greatest values (3.61% and 2.1%, respectively). Laggard chromosomes were only detected in five genotypes with low frequencies: ‘AI’, Cr7 x AI F^1^ cross, ‘Com1’ cv., D331 x Com1 F_1_ cross, and Cr7 x Com1 F_2_ cross. Micronuclei had the lowest frequency of all mitotic aberrations detected in this experiment. It was found in only Cr7 x AI F_1_ cross and ‘Com1’ cv., and at extremely low levels (0.14% and 0.12%, respectively).

**Table 8:** Proportions of total mitotic abnormalities derived from the root tips of six crosses in F_1_ and F_2_ produced from crossing between five parental genotypes; Cream 7 cv. ‘Cr7’, Colossus cv. ‘Col’, and Dokki 331 cv. ‘D331’ genotypes as female parents and Asian Introduction ‘AI’ and Commercial 1 ‘Com1’ genotypes as male parents.

Genotypes	Number of examined cells	Bridges%	Outside%	Stickiness%	Laggards%	Micro nuclei%	Total Abnormalities%
**Parental parents**							
Asian Introduction (AI)	1165	0.00	1.19	0.00	0.74	0.00	1.93
Colossus (Col)	1108	1.96	0.00	0.00	0.00	0.00	1.96
Commercial 1 (Com1)	1153	0.00	0.88	1.01	1.01	0.12	2.90
Cream 7 (Cr7)	1046	0.00	2.16	3.61	0.00	0.00	5.77
Dokki 331 (D331)	1171	0.00	0.76	1.95	0.00	0.00	2.71
**Crosses (F_1_)**							
Col x AI	1172	0.00	0.72	0.71	0.00	0.00	1.43
Col x Com1	1234	0.98	0.98	0.48	0.00	0.00	2.44
Cr7 x AI	1151	1.11	1.68	1.68	1.11	0.14	5.57
Cr7 x Com1	1220	1.15	0.00	0.68	0.00	0.00	1.83
D331 x AI	1182	0.81	0.90	0.00	0.00	0.00	1.71
D331 x Com1	1210	0.93	2.66	2.10	0.93	0.00	6.61
**Crosses (F_2_)**							
Col x AI	1364	0.51	0.42	0.00	0.00	0.00	0.93
Col x Com1	1267	0.00	1.21	0.85	0.00	0.00	2.07
Cr7 x AI	1359	1.90	0.95	0.85	0.00	0.00	3.71
Cr7 x Com1	1455	2.06	0.00	1.96	0.98	0.00	5.00
D331 x AI	1347	0.00	0.00	1.01	0.00	0.00	1.01
D331 x Com1	1383	1.83	0.00	0.00	0.00	0.00	1.83
LSD_0.05_		2.44	2.87	2.79	1.14	0.02	5.01

## Discussion

Cowpea’s narrow base of genetic diversity can be attributed to its self-pollinating nature, evolution from limited wild germplasm, and extremely minimal gene transfer between wild and cultivated varieties. Better variety breeding and selection is an important long-term technique to fighting the problem of low yield in arid or semi-arid regions (Zaki and Radwan, 2022). One of the most efficient traditional breeding methods is germplasm development and evaluation of promising varieties for adaptation and production stability (Tarawali et al., 2002; Singh, 2012). Breeding for consistent production would also require testing crop varieties in a wide range of environments both within and outside of regions, to identify superior genotypes with broad or specialized adaptation due to genotype x environment interactions (Nwosu and Awa, 2013). Summer season is especially challenging in Egypt and other African countries because of high temperatures combined with drought and other stressors. As a result, it will have a detrimental impact on vegetable productivity, quality, and production costs. Cowpea, being a major food in these regions, is thus introduced, particularly during the hot summer season, to provide fresh green vegetables all year (Reda et al., 2016). Introducing cultivars from other countries and planting them for evaluation to select superior cultivars is one of the necessary steps involved in the breeding for a new desired plant species (Animasaun et al., 2015; EL-Ameen, 2018). Varietal adaptation may vary dramatically among environments (Kaya et al., 2002; Eric et al., 2018). Meanwhile, the present study aims, *via* its crossings with introduced cultivars, such as ‘Col’ cv. and ‘AI’, to improve the performance of local commercial cultivars, such as ‘Cr7’ cv., ‘D331’ cv., and ‘Com1’. Plant breeders attempt to develop varieties that reduce the genotype’s adverse climatic interactions, varieties that can control their developmental processes so that high yields of high-quality food are produced (Singh et al., 1997; Animasaun et al., 2015).

The purpose of the current study was to determine the diversity of morphological, floral, pod, seed, and yield characteristics, as well as cytological analyses, among five distinct cowpea genotypes and six developed crosses from two generations apart, F_1_ and F_2_. The chosen genotypes and resulting crosses were very variable. Govindaraj et al. (2015) underlined the significance of genetic diversity as the lifeline of genetic improvement, whereas Meena et al. (2017) stated that the degree of genetic variability in the breeding population is dependent on the development of high yielding varieties.

In terms of the examined morphological, floral, pods and seeds traits, there were statistically significant variations amongst the genotypes for the most of traits. ‘AI’ had the longest pod length, while ‘D331’ cv. had the smallest length. A plant’s seed yield is significantly connected to the number of pods, the number of seeds per pod, and the weight of the pod (Oladejo et al., 2011). The findings of Jaydeep and Srinivasan (2011) demonstrated that the length of pods is a changeable trait that may be entirely or partially controlled by plant breeding. In general, heritability values are high for pod length (Diriba et al., 2014a and Diriba et al., 2014b; Sabale et al., 2018). F_1_ of D331 x AI cross produced the tallest shoot and the largest pod length, whereas F_2_ of Col x AI cross produced the longest pod length. Col x Com1 cross had the greatest peduncle diameter, and the most seeds per pod in F_1_ and F_2_. Cr7 x Com1 cross in F_1_ and F_2_ offspring surpassed the other crosses by having a taller peduncle than the better-parent and producing the most peduncle. F_2_ of Col x Com1 cross generated the best results and had the fewest ovules that were abortive per pod. In regards of seeds weight/pod, Col x Com1 cross was also superior in both generations. For seeds weight per plant, F_1_ of D331 x AI cross had the highest value and outperformed the parental genotypes as well as the other crosses in F_1_. That to say, crossing with ‘Col’, ‘AI’ and ‘Com1’ genotypes generally helped to improve the performance of the local varieties, ‘D331’ and ‘Cr7’. This demonstrates that this population might be exploited to develop a breeding program that would result in more productive progeny with more seeds, longer pods, and taller plants (Ajayi et al., 2014a and Ajayi et al., 2014b; Arup et al., 2014). This research has significance for the possibility for genetic development in the breeding program. The capability to select superior genotypes is entirely dependent on the genetic diversity of the collection of varieties, which is a function of additive variance. Important for the selection of possible genotypes is the presence of genetic variation in progenitors (Krause et al., 2012; Sabale et al., 2018).

Furthermore, the current study found a larger percentage of genotypic variance (GV) to total phenotypic variance (PV) in cowpea for selected agronomically significant variables. Other studies (Allen and Allen, 1981; Singh and Rachie, 1985; Damarany, 1994a; Ishiyaku et al., 2005) found a substantial fraction of GV in cowpea that contributed significantly to PV. The GV in F_1_ and F_2_ was greater than the PV in the five parents for the number of pods/plant which showed high H % values. Omoigui et al. (2006) found a H of 20% for the number of pods/plant, which was very low compared to the current estimate. In contrast to these findings, Singh and Rachie (1985) and Damarany (1994b) calculated the H of the number of pods/plant to be 53% and 86%, respectively. Parental genotypes, as well as F_1_ and F_2_ crosses, had high H % and genetic advance mean (GAM) for pod length and number of seeds/pod. Nevertheless, the five parents showed high genotypic coefficient of variance (GCV %), phenotypic coefficient of variance (PCV %), H %, and GAM of the number of aborted ovules/pod. Except for the F_1_ crosses, D331 x Com1 and Cr7 x AI, which had moderate values for H % for pod weight, all crossings in F_1_ and F_2_ exhibited high values for H % for the trait. All F_1_ and F_2_ crosses, on the other hand, produced moderate to high PCV % and GCV % of seeds weight/pod. These differences show variability as a result of additive and non-additive gene effects, emphasizing the possibility of developing novel varieties or hybrids (Silva et al., 2004; Adewale et al., 2011; Manggoel et al., 2012; Ogunkanmi et al., 2013; Lal et al., 2014). Carvalho et al. (2012) discovered similar results in cowpea for 100-seed weight, number of seeds/pod, and yield, whereas Kimani and Derera (2009) reported the same flowering time, number of seeds/pod, and 100-seed weight in common bean, indicating the presence of selection improvements.

In addition, the existing research investigated the cytological performance of produced crosses and parental genotypes to identify new prospective candidates for cowpea breeding programs. The same normal chromosomal number (2n = 22) was found in all cowpea genotypes and crosses studied. The majority of the genotypes/crosses had significantly different mitotic index (MI) overall numbers. Few genotypes showed a high prevalence of total mitotic chromosomal abnormalities. Mitotic abnormalities such as lagging chromosomes, chromosomal bridges, outside chromosomes, stickiness, and micronuclei were seen in all genotypes. The chromosomal number found in this study agrees with previous findings of 2n = 22 for *V. unguiculata* and some allied wild species (Damayanti et al., 2010; Shambhu, 2013). By investigating mitotic characteristics like as mitotic index and mitotic abnormalities, a tentative portrait of cytogenetic differences among genotypes/crosses of interest might be produced. The mitotic index is a variable that may be used to assess the frequency of cellular division (Marcano et al., 2004; Leme and Marin-Morales, 2009). The means of the mitotic index (MI) were substantially different amongst genotypes, owing to large variability in the percentage values of mitotic phases. This might be due to changes in mitotic genetic regulatory systems (cell cycle programs) and/or the number of somatic mutations (Yasuhara and Shibaoka, 2000). This study demonstrated that assessing cowpea genotypes and produced crosses under realistic growth circumstances may objectively reveal the differential contributions of genotype through selection. In addition, the approach provides adequate estimates of variance components and heritability of certain traits, highlighting the significant impact of genotypic variation on traditional breeding. There is sufficient evidence to imply a substantial relationship between the ranks of the variance components’ magnitudes.

## Conclusion

Six crosses were developed in two generations apart using five different parental genotypes: ‘AI’, ‘Col’, ‘Com1’, ‘Cr7’, and ‘D331’. When examined under different crossings in F_1_ and F_2_, the study indicated that there is a substantial degree of genotypic variability across the variables investigated in cowpea parental genotypes. Genotypic variance was greater than phenotypic variance for the number of pods/plant, pod length, number of seeds/pod, number of aborted ovules/pod, fresh pod weight, seeds weight/pod, and seeds yield/plant. Except for seeds weight/pod in ‘D331’ cv. and F_2_ of Col x Com1, genotypes/crosses showed high H % for all variables tested. F_2_ plants had a greater H % than F_1_ plants and the genotypes of their parents. The produced crosses, Cr7 x Com1 and D331 x AI, have the potential for future genetic breeding research and are especially promising for yield and yield component selection. This might allow cowpea producers in Egypt and other similar regions to engage in strategic breeding and breeding trait modification.

## Data availability statement

The original contributions presented in the study are included in the article/**Supplementary Material**. Further inquiries can be directed to the corresponding author.

## Author contributions

HZ: conceptualization, data curation, formal analysis, investigation, methodology, project administration, software, supervision, validation, visualization, writing - original draft, and writing - review and editing. KR: data curation, formal analysis, investigation, methodology, visualization, writing - original draft, and writing - review and editing. All authors contributed to the article and approved the submitted version.

## Acknowledgments

The authors would like to express their gratitude to the Department of Genetics, Faculty of Agriculture, Minia University, El-Minia, Egypt, for technical assistance with cytological examination of cowpea genotypes.

## Conflict of interest

The authors declare that the research was conducted in the absence of any commercial or financial relationships that could be construed as a potential conflict of interest.

## Publisher’s note

All claims expressed in this article are solely those of the authors and do not necessarily represent those of their affiliated organizations, or those of the publisher, the editors and the reviewers. Any product that may be evaluated in this article, or claim that may be made by its manufacturer, is not guaranteed or endorsed by the publisher.

## Abbreviations

‘AI’, Asian Introduction; ‘Col’, Colossus cv.; 'Com1', Commercial 1; 'Cr7', Cream 7 cv.; 'D331', Dokki 331 cv.; 'PV', Phenotypic Variance; 'GV', Genotypic Variance; 'PCV', Phenotypic Coefficient of Variance; 'GCV', Genotypic Coefficient of Variance; 'H', Heritability; 'GAM', Genetic Advance Mean; 'MI', Mitotic Index.
