# Supplementary material for: Estimates of genotypic and phenotypic variance, heritability, and genetic advance of horticultural traits in developed crosses of cowpea (Vigna unguiculata [L.] Walp)
Source: Front Plant Sci. 2022 Sep 27;13:987985. doi: 10.3389/fpls.2022.987985 (PMC9551400; doi:10.3389/fpls.2022.987985)
Supplement: Supplementary file 1 [file DataSheet_1.docx]

Supplementary Material

**Supplementary Table 1.** Distribution of particle size and chemical properties of the experimental site soil.

| **Particle size distribution*** | **Values** |
| --- | --- |
| Sand, % | 49.5 |
| Silt, % | 23.8 |
| Clay, % | 26.7 |
| Texture | Sandy clay loam |
| **Chemical characteristics** | |
| pH | 7.60 |
| E.C.E (ds/m) | 1.20 |
| HCO_3_ | 2.41 |
| Cl | 2.20 |
| Mg^+2^ | 1.90 |
| Na ^+^ | 6.20 |
| K^+^ | 0.21 |
| N (mg/L) | 62.4 |
| P (mg/L) | 9.20 |
| K (mg/L) | 356 |
| SO4^-2^(mg/L) | 6.60 |
| Ca^+2^ (mg/L) | 4.30 |

*Soil was sampled during the studied seasons of 2016, 2017 and 2018 at a depth of 0.0-30 cm and the average data were obtained. Extract was (1:1) ml/100g soil.

**Supplementary Table 2**. Genotypic variance (GV), phenotypic variance (PV), genotypic coefficient of variance (GCV%), phenotypic coefficient of variance (PCV%), heritability (H%), and genetic advance mean (GAM) of morphological, and flower traits of the five parental genotypes; local commercial cultivars, i.e., Cream 7 cv. ‘Cr7’, Dokki 331 cv. ‘D331’, and introduced cultivars, i.e., Colossus ‘Col’ and Asian Introduction ‘AI’. Another cultivar, Commercial 1 ‘Com1’, was collected from the local market for its seed’s quality characteristics.

| **Parental genotypes** | **Traits*** | **GV** | **PV** | **GCV %** | **PCV %** | **H %** | **GAM** |
| --- | --- | --- | --- | --- | --- | --- | --- |
| AI | SL | 238.23 | 233.35 | 8.356 | 8.27 | 97.95 | 16.86 |
|  | NB/P | 0.54 | 0.31 | 25.43 | 19.23 | 57.16 | 29.97 |
|  | PeL | 30.18 | 28.44 | 22.51 | 21.85 | 94.24 | 43.71 |
|  | NPe/P | 15.73 | 14.47 | 21.79 | 20.90 | 92.02 | 41.32 |
| Col cv. | SL | 639.28 | 631.29 | 18.16 | 18.05 | 98.74 | 36.94 |
|  | NB/P | 0.76 | 0.49 | 21.36 | 17.07 | 63.88 | 28.11 |
|  | PeL | 51.32 | 49.05 | 24.61 | 24.06 | 95.58 | 48.47 |
|  | NPe/P | 158.1 | 154.2 | 45.24 | 44.66 | 97.48 | 90.85 |
| Com1 | SL | 450.66 | 443.95 | 15.84 | 15.72 | 98.51 | 32.14 |
|  | NB/P | 2.27 | 1.80 | 27.44 | 24.40 | 79.06 | 44.68 |
|  | PeL | 21.71 | 20.23 | 18.02 | 17.40 | 93.21 | 34.61 |
|  | NPe/P | 54.22 | 51.89 | 24.54 | 24.01 | 95.70 | 48.39 |
| Cr7 cv. | SL | 247.61 | 242.63 | 14.77 | 14.62 | 97.99 | 29.82 |
|  | NB/P | 8.71 | 7.77 | 39.88 | 37.68 | 89.28 | 73.35 |
|  | PeL | 57.23 | 54.84 | 22.78 | 22.30 | 95.82 | 44.97 |
|  | NPe/P | 289.5 | 284.1 | 43.63 | 43.22 | 98.14 | 88.20 |
| D331 cv. | SL | 1143.9 | 1133.2 | 25.39 | 25.27 | 99.06 | 51.81 |
|  | NB/P | 1.15 | 0.81 | 16.79 | 14.11 | 70.58 | 24.42 |
|  | PeL | 31.89 | 30.10 | 17.64 | 17.14 | 94.39 | 34.30 |
|  | NPe/P | 224.7 | 220.0 | 30.65 | 30.33 | 97.89 | 61.81 |

*Shoot length (SL), number of branches/plant (NB/P), peduncle length (PeL), number of peduncles/plant (NPe/P)

**Supplementary Table 3.** Genotypic variance (GV), phenotypic variance (PV), genotypic coefficient of variance (GCV%), phenotypic coefficient of variance (PCV%), heritability (H%), and genetic advance mean (GAM) of morphological, and flower traits of the six crosses in F_1_ and F_2_ produced from crossing between five parental genotypes; Cream 7 cv. ‘Cr7’, Colossus cv. ‘Col’, and Dokki 331 cv. ‘D331’ genotypes as female parents and Asian Introduction ‘AI’ and Commercial 1 ‘Com1’ genotypes as male parents.

| **Crosses** | **Traits*** | **GV** | | **PV** | | **GCV %** | | **PCV %** | | **H %** | | **GAM** | |
| --- | --- | --- | --- | --- | --- | --- | --- | --- | --- | --- | --- | --- | --- |
|  |  | **F_1_** | **F_2_** | **F_1_** | **F_2_** | **F_1_** | **F_2_** | **F_1_** | **F_2_** | **F_1_** | **F_2_** | **F_1_** | **F_2_** |
| Col  x  AI | SL | 15.70 | 1311.7 | 10.17 | 1306.0 | 2.14 | 19.79 | 1.72 | 19.74 | 64.80 | 99.56 | 2.85 | 40.59 |
|  | NB/P | 1.20 | 1.5 | 0.71 | 1.31 | 17.95 | 25.57 | 13.81 | 23.87 | 59.16 | 87.12 | 21.88 | 45.91 |
|  | PeL | 83.0 | 61.89 | 78.9 | 60.65 | 30.37 | 21.09 | 29.62 | 20.87 | 95.09 | 97.99 | 59.50 | 42.57 |
|  | NPe/P | 9.20 | 55.26 | 7.84 | 54.09 | 7.71 | 25.9 | 7.12 | 25.62 | 85.26 | 97.87 | 13.55 | 52.22 |
| Col  x  Com1 | SL | 1984.5 | 927.67 | 1953 | 922.86 | 20.20 | 16.78 | 20.04 | 16.73 | 98.41 | 99.48 | 40.95 | 34.38 |
|  | NB/P | 8.00 | 1.74 | 6.00 | 1.53 | 31.42 | 26.92 | 27.21 | 25.25 | 75.00 | 87.99 | 48.55 | 48.79 |
|  | PeL | 2.00 | 50.08 | 1.00 | 48.96 | 3.36 | 19.23 | 2.38 | 19.01 | 50.00 | 97.76 | 3.46 | 38.73 |
|  | NPe/P | 98.00 | 39.25 | 91.00 | 38.26 | 17.99 | 21.53 | 17.34 | 21.25 | 92.85 | 97.47 | 34.42 | 43.23 |
| Cr7  x  AI | SL | 209.65 | 1447.4 | 205.47 | 1441.4 | 11.80 | 20.77 | 11.68 | 20.73 | 98.00 | 99.58 | 23.82 | 42.62 |
|  | NB/P | 1.35 | 2.45 | 1.02 | 2.21 | 23.28 | 29.03 | 20.19 | 27.53 | 75.22 | 89.91 | 36.08 | 53.78 |
|  | PeL | 28.34 | 61.89 | 26.80 | 60.65 | 20.63 | 19.96 | 20.06 | 19.76 | 94.57 | 97.99 | 40.20 | 40.30 |
|  | NPe/P | 34.69 | 78.91 | 32.99 | 77.51 | 19.56 | 23.5 | 19.08 | 23.29 | 95.10 | 98.21 | 38.33 | 47.55 |
| Cr7  x  Com1 | SL | 3653.1 | 831.52 | 3634.0 | 826.96 | 38.15 | 18.77 | 38.05 | 18.72 | 99.47 | 99.45 | 78.19 | 38.46 |
|  | NB/P | 7.28 | 2.07 | 6.43 | 1.84 | 34.61 | 27.18 | 32.52 | 25.64 | 88.28 | 89.01 | 62.94 | 49.85 |
|  | PeL | 130.3 | 118.0 | 126.7 | 116.29 | 21.99 | 27.29 | 21.68 | 27.29 | 97.22 | 98.54 | 44.05 | 55.40 |
|  | NPe/P | 1715.1 | 124.0 | 1702.0 | 122.2 | 65.32 | 32.95 | 65.07 | 32.71 | 99.23 | 98.58 | 133.53 | 66.91 |
| D331  x  AI | SL | 1357.3 | 1580.6 | 1340.8 | 1574.3 | 25.16 | 24.27 | 25.01 | 24.22 | 98.78 | 99.60 | 51.21 | 49.80 |
|  | NB/P | 2.70 | 1.57 | 1.96 | 1.37 | 24.16 | 23.67 | 20.61 | 22.13 | 72.77 | 87.42 | 36.22 | 42.60 |
|  | PeL | 68.92 | 61.96 | 65.21 | 0.723 | 34.02 | 23.28 | 33.09 | 23.05 | 94.61 | 97.99 | 66.31 | 47.01 |
|  | NPe/P | 264.3 | 42.04 | 257.0 | 41.02 | 32.90 | 24.01 | 32.45 | 23.72 | 97.24 | 97.56 | 65.92 | 48.26 |
| D331  x  Com1 | SL | 2.00 | 951.98 | 1.00 | 947.10 | 0.60 | 23.05 | 0.42 | 23.00 | 50.00 | 99.48 | 0.62 | 47.25 |
|  | NB/P | 12.5 | 1.08 | 10.0 | 0.92 | 41.59 | 21.72 | 37.20 | 20.00 | 80.00 | 84.82 | 68.54 | 37.96 |
|  | PeL | 8.00 | 60.09 | 6.00 | 58.86 | 7.54 | 22.02 | 6.53 | 21.79 | 75.00 | 97.95 | 11.65 | 44.44 |
|  | NPe/P | 11.00 | 57.19 | 10.00 | 55.99 | 9.77 | 21.79 | 8.33 | 21.56 | 77.00 | 97.90 | 56.15 | 43.95 |

*Shoot length (SL), number of branches/plant (NB/P), peduncle length (PeL), number of peduncles/plant (NPe/P)
